# Structural brain network measures in elderly patients with cerebral small vessel disease and depressive symptoms

**DOI:** 10.1186/s12877-022-03245-7

**Published:** 2022-07-09

**Authors:** Yumeng Gu, Ping Zhao, Wenjun Feng, Xiaoshuang Xia, Xiaolin Tian, Yu Yan, Xiaowen Wang, Decheng Gao, Yanfen Du, Xin Li

**Affiliations:** 1 Department of Neurology, Second Hospital of Tianjin Medical University, No.23, Pingjiang Road, Hexi District, Tianjin, 300211 China; 2 Department of Rehabilitation, Second Hospital of Tianjin Medical University, Tianjin, 300211 China

**Keywords:** Cerebral small vessel disease, Depression, Diffusion tensor imaging, Structural network, Tract-based spatial statistics

## Abstract

**Objectives:**

To investigate the relationship between diffusion tensor imaging (DTI) indicators and cerebral small vessel disease (CSVD) with depressive states, and to explore the underlying mechanisms of white matter damage in CSVD with depression.

**Method:**

A total of 115 elderly subjects were consecutively recruited from the neurology clinic, including 36 CSVD patients with depressive state (CSVD+D), 34 CSVD patients without depressive state (CSVD-D), and 45 controls. A detailed neuropsychological assessment and multimodal magnetic resonance imaging (MRI) were performed. Based on tract-based spatial statistics (TBSS) analysis and structural network analysis, differences between groups were compared, including white matter fiber indicators (fractional anisotropy and mean diffusivity) and structural brain network indicators (global efficiency, local efficiency and network strength), in order to explore the differences and correlations of DTI parameters among the three groups.

**Results:**

There were no significant differences in terms of CSVD burden scores and conventional imaging findings between the CSVD-D and CSVD+D groups. Group differences were found in DTI indicators (*p* <  0.05), after adjusting for age, gender, education level, and vascular risk factors (VRF), there were significant correlations between TBSS analysis indicators and depression, including: fractional anisotropy (FA) (*r* = − 0.291, *p* <  0.05), mean diffusivity (MD) (*r* = 0.297, *p* < 0.05), at the same time, between structural network indicators and depression also show significant correlations, including: local efficiency (E_Local_) (*r* = − 0.278, *p* < 0.01) and network strength (*r* = − 0.403, *p* < 0.001).

**Conclusions:**

Changes in FA, MD values and structural network indicators in DTI parameters can predict the depressive state of CSVD to a certain extent, providing a more direct structural basis for the hypothesis of abnormal neural circuits in the pathogenesis of vascular-related depression. In addition, abnormal white matter alterations in subcortical neural circuits probably affect the microstructural function of brain connections, which may be a mechanism for the concomitant depressive symptoms in CSVD patients.

## Introduction

With the gradual increase in the number of elderly people in China, cardiovascular and cerebrovascular diseases are frequent, and the incidence of cerebral small vessel disease (CSVD) is also on the rise. CSVD is a highly prevalent age-related disease. In China, lacunar infarction (LI) caused by CSVD accounts for 25 to 50% of ischemic stroke, which is higher than the occurrence in Western countries [1]. The prevalence of white matter hyperintensity increases from 50 to 95% between the ages of 50 and 80 [2]，cerebral microbleeds are 24% [3], and enlarged perivascular space (EPVS) is as high as 79.9% [4]. CSVD is an important cause of vascular-related depression [5, 6]. Given that the underlying pathogenesis remains controversial [7], multimodal neuroimaging is usually used to detect lesions that lead to CSVD. There are many recognized CSVD structural markers on MRI [8], including WMH, LI, CMBs, and EPVS. These features of CSVD imaging are interrelated, and their correlation with depression has been indicated [9, 10].

With the continuous development of multimodal neuroimaging technology, the understanding of brain information processing tends to create a new approach to the interconnected holistic brain network analysis. Currently, many studies have shown that brain network connections in CSVD patients are disrupted [11–13], and the diffusion tensor imaging (DTI) is a method used to study white matter fiber disconnection damage and explore damage to structural connections of brain regions [14]. White matter microstructural damage caused by CSVD destroys the integrity of white matter fibers and damages the nerve fiber network, resulting in symptoms such as psychomotor retardation, hypomotility, low mood, and cognitive decline [15]. The damage of the brain white matter microstructure caused by CSVD is related to psychomotor retardation, hypomotility, low mood, and cognitive decline [15]. However, whether these new findings can be identified in CSVD patients with depressive symptoms has not been determined.

Therefore, we aimed to explore the relationship between white matter microstructure and depression in patients with CSVD by using different DTI measurements. The main DTI parameters evaluated were fractional anisotropy (FA), mean diffusion (MD), axial diffusion (AD), and radial diffusion (RD). We chose FA and MD to represent fiber tract measurements, as they are the most commonly used indicators. By comparing tract-based spatial statistics (TBSS) analysis indicators and structural brain network measures in CSVD patients, we hope to provide some evidence for the potential mechanism of early depression in patients with CSVD. In addition, we aimed to search for more downstream neuroimaging markers in patients with CSVD-induced depression.

## Methods

### Participants

We recruited 115 right-handed elderly subjects from the Neurology Department of the Second Hospital of Tianjin Medical University. The inclusion criteria of CSVD patients were as follows: (1) 50 to 80 years old; (2) at least 6 years of education; (3) Meet Staals scoring criteria [16]; (4) Informed consent signed by participants. All CSVD patients underwent overall psychological function through Montreal Cognitive Assessment (MoCA). Patients were assessed for depression using the 17 item Hamilton Depression Rating Scale (HAMD-17). Patients with a HAMD-17 score of 7–17 were classified as CSVD with depressive state (CSVD+D), while those with a score below 7 were classified as CSVD without depressive state (CSVD-D) [17–19].

Exclusion criteria: (1) severe physical disability; (2) WMH due to non-vascular dysfunction; (3) Cognitive impairment; (4) Clinical dementia score (CDR) < 0.5; (5) MoCA score < 26; (6) No stenosis of intracranial and extracranial vascular; (7) Cardiogenic cerebral embolism; (9) Alcohol or drug abuse disorder or serious mental disorder; (10) Inability to cooperate with evaluation or MRI.

Forty-five controls (Con) were included from Tianjin, China. The inclusion criteria of NC are as follows: (1) No clinical stroke history; (2) at least 6 years of education; (3) No history of major diseases of liver, heart, lung and other important organs; (4) Fazekas grade ≤ 1 and MRI scan showed no other significant abnormalities; (5) HAMD-17 score < 8; (6) Cognitive assessment test were normal; (7) No vascular risk factors such as hypercholesterolemia, diabetes, hypertension, and smoking evidence.

During the course of the study, 3 CSVD patients were excluded for personal reasons, 2 controls and 2 CSVD patients for visible head movement during MRI. Therefore, 36 CSVD+D patients, 34 CSVD-D patients and 45 Con were included. All subjects gave written informed consent, and this study was approved by the institutional review board of the second hospital of Tianjin Medical University.

### MRI acquisition

MRI scans were performed on a 3.0 T scanner (GE HealthCare, USA). T1 images parameters: TR = 8.2 ms, TE = 3.2 ms, TI = 450 ms, slice thickness = 1.0 mm, gap = 0, flip angle = 12°, FOV = 256 × 256 mm^2^, slice number = 166; T2 FLAIR images parameters: TE = 150 ms, TR = 9075 ms, TI = 2250 ms, FOV = 256 mm2, number of slices = 160; Diffusion-weighted imaging was acquired in the anterior-to-posterior phase-encoding direction, and b-values for non-zero gradient volumes were 1000 s/mm^2^ along 32 gradient directions. Acquisition of each diffusion-weighted image was completed with a gradient-free image, and b-value for the reference volumes were 0. Images scanned in the opposite phase-encoding direction were also acquired to correct for distortions caused by susceptibility in the scans. The DTI sequences parameters were as follows: TR = 8000 ms, TE = 88.4 ms, matrix = 128 × 128, FOV = 256 × 256 mm, NEX = 1, slice thickness = 2 mm, gap = 0 and number of slices  =  75.

### Grading and evaluation of CSVD lesions on MRI

The total burden of CSVD imaging was reflected using a well validated CSVD burden scoring scale, which is an ordinal scoring scale of 0–4 points [16]. One point when there is one or more lacunar lesions (LI) on MRI; one point when there are deep microbleeds (CMBs); one point when 3 scores on the Fazekas scale for periventricular white matter hyperintensities (WMHs) and (or) 2–3 scores on the Fazekas scale of deep WMHs; one point when the grade of enlarged perivascular space (EPVs) in basal ganglia is grade 2–3 [16]. The number of CMBs was scaled by Microbleed anatomical grading scale (MARS) [20]. Maclulich’s method was used to record and score EPVS [21]. The above scores were rated by two experienced neurologists blinded to clinical data. The CSVD burden scores and conventional imaging findings between the CSVD-D and CSVD+D groups were compared.

### MRI data preprocessing analysis

#### Structural data

Structural images were preprocessed using the CAT12 toolbox available in the Statistical Parametric Mapping 12 package (SPM12). The following steps were used through MATLAB 2019a: normalized T1 images to template space and segmented images into gray matter, white matter, and cerebrospinal fluid; visual inspection; the total intracranial volume was evaluated; check for data homogeneity; the Gaussian kernel of full width at half maximum 6 mm (FWHM = 6 mm) was used. The “brain volume/ intracranial volume (ICV) x 100%” was used to display WMHs, gray and white matter volume [22].

#### DTI measures

DTI images were pre and post processed using FSL software and analyzed based on TBSS [23]. Skeleton threshold was set to less than 0.2. Global FA and MD values were computed by averaging the FA/MD values across the whole skeleton. The skeletonized FA or MD maps were entered into a voxel group level analysis. Statistical significance was assessed using the FSL permutation test. As the statistical modeling of voxel wise statistical analysis, the general linear model (GLM) was performed. Voxel-based pairwise group comparisons of CSVD-D versus CSVD+D, Con versus CSVD+D, and Con versus CSVD-D were performed using GLM. The covariates included sex, age, education, and vascular risk factors (VRF). The significance level of the two DTI parameters (FA, MD) is *p*<0.05 (5000 permutations, strong control of familywise error [FWE] correction for multiple comparisons correction using the threshold-free cluster enhancement) [24]. To illustrate the difference between the groups, displayed using the JHU white matter tractography atlas.

#### Brain network construction

Using the Pipeline of the Analyze Brain Diffusion Image Toolkit (PANDA) to preprocess the raw DTI data [25]. Using the automated anatomical labeling (AAL) 90 atlas [26]. The Fiber Assignment Continuous Tracking (FACT) algorithm was used to generate whole brain white matter fiber reconstructions. The number of streamlines is the surrogate measure of the number of fibers [27]. Streamlines terminate if the fiber is switched on at an angle > 45 ° or encounters a voxel with FA < 0.2 [28]. The structured brain network was ultimately structured by 90 for each subject 90 × 90 fiber number (FN) weighted matrix construction. Using the graph theoretic network analysis toolbox (GRETNA) [29], global topology metrics were calculated. Different rarefaction thresholds ranging from 0.1 to 0.3 at 0.01 intervals. The topological property parameters analyzed in this study include: global efficiency (E_Global_), local efficiency (E_Local_), network strength and small world properties. The network strength was defined as the overall accumulation of fiber number on all nodes [30]. The regional efficiency of each node is a measure of its connectivity to all other nodes in the network [31]. Finally, a 3D brain map was visualized using the BrainNet Viewer (http://www.nitrc.org/projects/bnv/) [32] to intuitive display the position of damaged nodes after Bonferroni correction.

### Statistical methods

Analyses were performed with SPSS version19.0 (IBM Corp., Armonk, NY, USA), baseline data were analyzed by one-way analysis of variance (ANOVA). Normally distributed measurement data are expressed as ^−^x ± *s*. The non-normally distributed continuous variables were expressed as median (interquartile ranges, IQR) and were compared using the Wilcoxon rank-sum test. The categorical data were expressed as numbers with percentages and comparisons between groups were performed using the χ^2^ test. Post hoc test was used for comparisons between groups, and Bonferroni correction was used for multiple comparisons. Partial correlation analysis was used to assess the relationship between Hamilton Depression Score and DTI measures in patients with CSVD after adjusting for age, sex, education, and VRF. Two-tailed *p*-value < 0.05 was considered statistically significant.

## Result

### Demographic data and clinical measurements

Among the subjects (*n* = 115), 70 subjects were diagnosed with CSVD, of which 36 CSVD patients were diagnosed with CSVD+D group, the remaining 34 CSVD patients were CSVD-D group, and 45 subjects were Con group. The baseline data are listed in Table 1. There were no significant differences in age, gender, education and MoCA scores among the three groups (*p* > 0.05). There were no significant differences in VRF between the CSVD-D and CSVD+D groups (*p* > 0.05). The HAMD scores of CSVD+D group were the highest among the three groups, representing the most severe depression. HAMD scores were significantly lower in Con group compared to CSVD-D and CSVD+D groups.

**Table 1:** Demographic and clinical characteristics

	Con *n* = 45	CSVD−D *n* = 34	CSVD+D *n* = 36	*p*-value
**Demographic factors**				
Male (%)	20 (44.4)	19 (55.9)	19 (52.8)	0.569
Age, mean (SD)	63.33 (6.03)	63.06 (5.22)	63.42 (6.53)	0.966
Education, mean (SD)	10.60 (2.42)	10.56 (2.14)	10.25 (2.64)	0.788
**Vascular risk factors**				
Hypercholesterolemia (%)	–	8 (23.5)	9 (25.0)	0.886
Diabetes Mellitus (%)	–	7 (20.6)	12 (33.3)	0.231
Hypertension (%)	–	17 (50.0)	20 (55.6)	0.642
Smoking (%)	–	17 (50.0)	17 (47.2)	0.816
Drinking (%)	–	15 (44.1)	16 (44.4)	0.978
**Neuropsychological tests**				
MoCA, mean (SD)	27.80 (1.47)	27.59 (1.58)	27.42 (1.38)	0.513
HAMD, mean (SD)	1.91 (0.99)	2.29 (1.61)	11.53 (2.66)	< 0.001^a,b,c^

Data represent number (percentage) and mean (standard deviation) for normally distributed data; SD, standard deviation; *p*-value < 0.05 was considered to be statistically significant

*Abbreviations*: *CSVD* Cerebral small vessel disease, *CSVD−D* CSVD without depression symptoms; *CSVD+D* CSVD with depression symptoms, *Con* Control group, *MOCA* Montreal Cognitive Assessment, *HAMD* Hamilton Depression Rating Scale; a–c: post hoc analysis revealed the source of ANOVA (a: Con vs. CSVD−D, b: Con vs. CSVD+D, and c: CSVD−D vs. CSVD+D)

### Neuroimaging findings in patients with CSVD

**Fig. 1:**
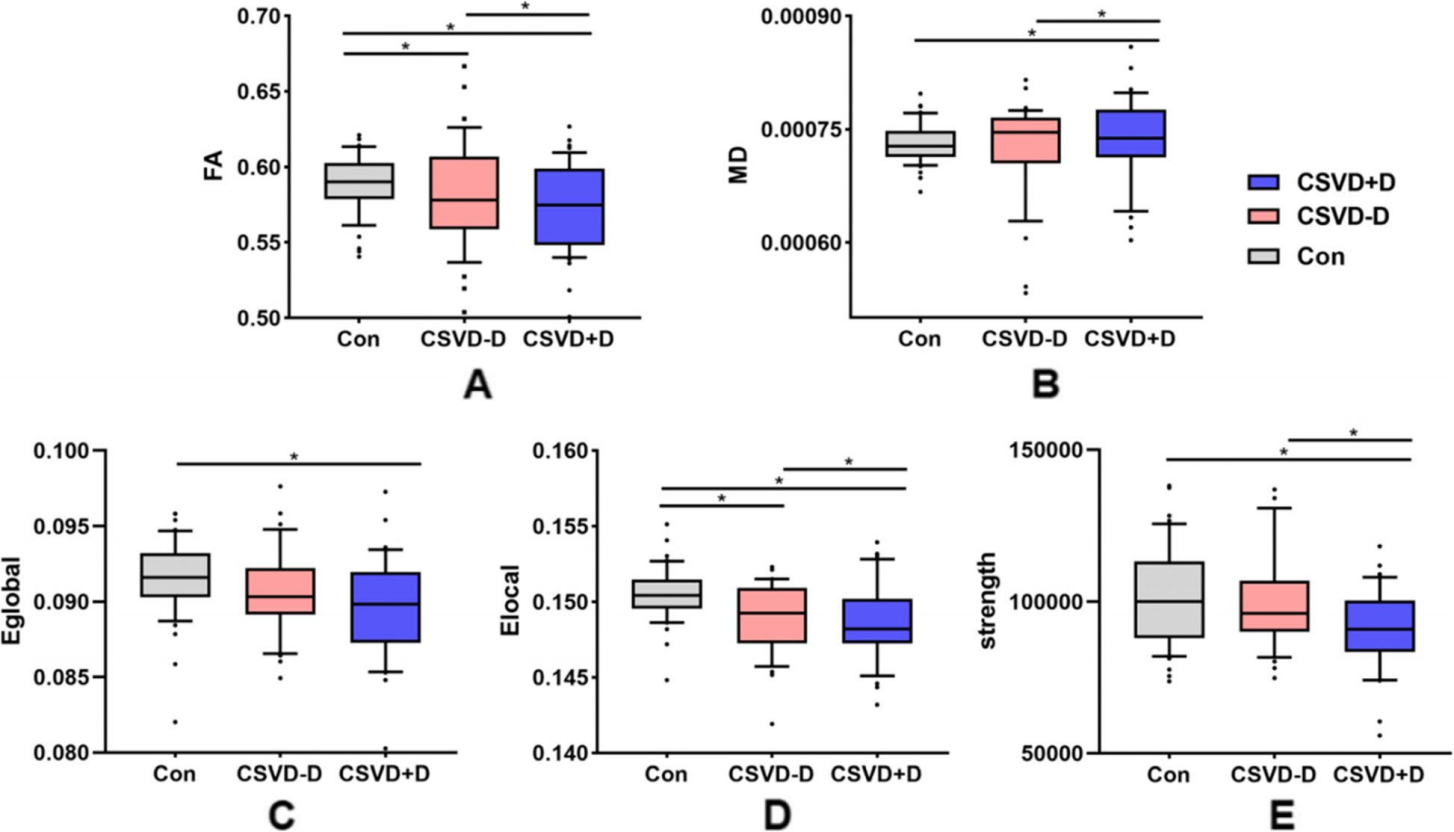
Group difference of DTI measures. (A–E) One-way ANOVA test was applied to conduct the group comparison of DTI measures, *p*-value < 0.05 was considered to be statistically significant. * represents *p*-value < 0.05 after Bonferroni correction. Abbreviations:CSVD−D, CSVD without depression symptoms; CSVD+D, CSVD with depression symptoms; Con, control group; FA, mean fractional anisotropy of the skeleton; MD, mean of mean diffusivity of the skeleton; Eglobal, global efficiency; Elocal, local efficiency

There were no significant differences in terms of CSVD burden scores between the CSVD-D and CSVD+D groups, imaging findings, and brain volume (as a percentage of total intracranial volume), and the results are shown in Table 2. The results of group comparisons of the DTI measurement indexes are shown in Fig. 1. FA, MD, E_Global_, E_Local_, and network strength showed significant differences among the three groups, further descriptive details can be found in Table 3. The values of FA, E_Local_ and network intensity were significantly lower in CSVD+D than those in CSVD-D and Con groups, E_Global_ showed significant difference between CSVD+D and Con groups. Meanwhile, MD was significantly higher in CSVD+D than that in CSVD-D and Con groups (Bonferroni corrected, *p* < 0.05).

**Table 2:** Neuroimaging findings of CSVD patients

	CSVD−D *n* = 34	CSVD+D *n* = 36	*p*-value
**Total CSVD Burden**			
CSVD score, median (IQR)	1 (1)	1.5 (1)	0.415
Grade 1, n (%)	23 (67.6)	18 (50.0)	
Grade 2, n (%)	8 (23.5)	12 (33.3)	
Grade 3, n (%)	3 (8.8)	5 (13.9)	
Grade 4, n (%)	0 (0)	1 (2.8)	
**Cerebrovascular lesions**			
WMH Fazekas 0–1, n (%)	9 (26.5)	4 (11.1)	0.099
WMH Fazekas 2–3, n (%)	25 (73.5)	32 (88.9)	
Lacunes occurrence, n (%)	12 (35.3)	18 (50.0)	0.214
CMBs occurrence, n (%)	8 (23.5)	9 (25.0)	0.886
EPVS occurrence, n (%)	3 (8.8)	2 (5.6)	0.596
**Neuroimaging measures**			
Total GMV, mean (SD)	32.81 (2.33)	32.74 (2.11)	0.896
Total WMV, mean (SD)	26.94 (3.21)	27.39 (2.56)	0.435
Total WMHV, mean (SD)	3.14 (1.95)	4.01 (2.27)	0.091

Data represent mean ± standard deviation or median (interquartile range). * means *p*-value < 0.05 was considered to be statistically significant

*Abbreviations*: *CSVD* Cerebral small vessel disease; *CSVD-D* CSVD without depression symptoms, *CSVD+D* CSVD with depression symptoms, *Con* Control group, *CMBs* Cerebral microbleeds, *WMH score* White matter hyperintensity score; *EPVS* Enlarged perivascular spaces, *TIV* Total intracranial volume, *Total GMV* gray matter volume as % of TIV, *Total WMV* White matter hyperintensity as % of TIV, *Total WMHV* White matter hyperintensity volume as % of TIV, *SD* Standard deviation, *IQR* Interquartile range

**Table 3:** DTI measures

	Con *n* = 45	CSVD−D *n* = 34	CSVD+D *n* = 36	*p*-value
**Whole-brain diffusion metrics**				
FA, mean (SD)	0.589 (0.011)	0.580 (0.021)	0.570 (0.016)	< 0.001^a,b,c^
MD*10^−3^, mean (SD)	0.731 (0.009)	0.727 (0.015)	0.738 (0.014)	0.002^b,c^
**Structual network measures**				
E_Global_*10^−3^,mean (SD)	91.745 (3.251)	90.045 (3.906)	88.388 (4.352)	< 0.001^b^
E_Local_*10^−3^, mean (SD)	150.521 (1.756)	149.015 (2.190)	147.665 (2.543)	< 0.001^a,b,c^
Network strength, mean (SD)	101,236.8 (16,367.089)	100,392.61 (17,512.795)	90,347 (13,532.756)	0.006^b,c^

Data represent mean ± standard deviation. *p*-value < 0.05 was considered to be statistically significant

*Abbreviations*: *CSVD-D* CSVD without depression symptoms, *CSVD+D* CSVD with depression symptoms, *Con* Control group, *FA* Mean fractional anisotropy of the skeleton, *MD* Mean of mean diffusivity of the skeleton, *E*_*Global*_ Global efficiency, *E*_*Local*_ Local efficiency

^a^represents significant difference between Con and CSVD−D

^b^represents significant difference between Con and CSVD+D

^c^ represents significant difference between CSVD−D and CSVD+D

### FA and MD of whole-brain Fiber tracts

**Fig. 2:**
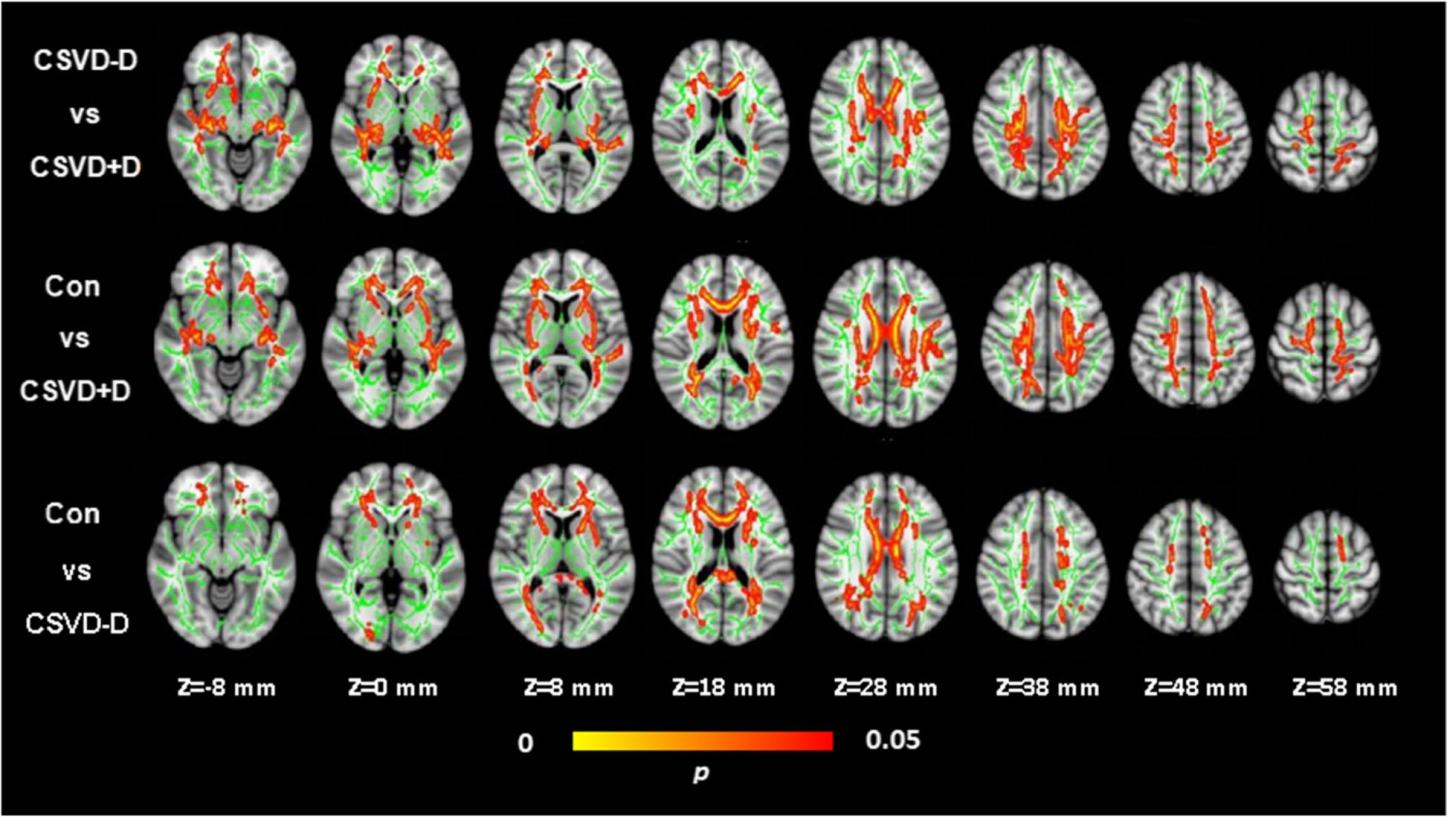
Voxel-wise Tract-Based Spatial Statistics differences in FA metrics between groups. Green represents the mean WM skeleton of all subjects. Top row: Red-yellow voxels (thickened for better visibility) represent the WM regions with decreased FA in the CSVD+D patients compared with CSVD-D subjects (FWE corrected, *p* < 0.05). Middlerow: Red-yellow voxels represent the WM regions with reduced FA in the CSVD+D group compared with control group (FWE corrected, *p* < 0.05). Bottom row: Red-yellow voxels represent the WM regions with reduced FA in the CSVD-D group compared with control group (FWE corrected, *p* < 0.05)

**Fig. 3:**
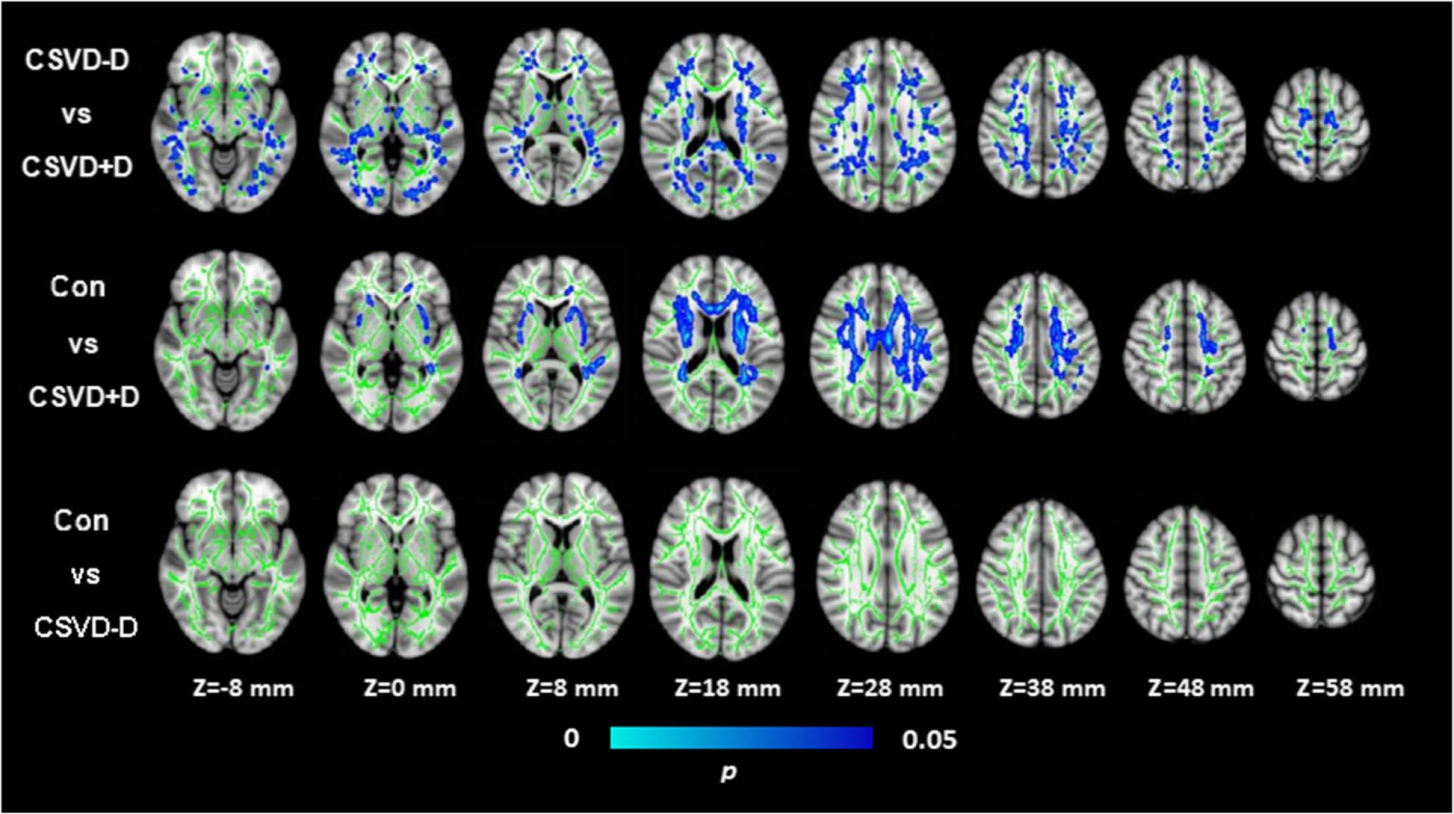
Voxel-wise Tract-Based Spatial Statistics differences in MD metrics between groups. Green represents the mean WM skeleton of all subjects. Top row: bule voxels (thickened for better visibility) represent the WM regions with increased MD in the CSVD+D patients compared with CSVD-D subjects (FWE corrected, *p* < 0.05). Middlerow: bule voxels represent the WM regions with increased MD in the CSVD+D group compared with control group (FWE corrected, *p* < 0.05). Bottom row: There are no significant WM microstructural differences between the CSVD-D patients and control subjects

For evaluating voxel WM integrity differences between each group pair using the TBSS method to analyze DTI parameters (Fig. 2 and Fig. 3). Compared with the CSVD-D group, the CSVD+D group showed significantly decreased FA values and significantly elevated MD values in a wide range of WM tracts. Compared with the Con group, the CSVD+D group showed significantly decreased FA values and significantly increased MD values in extensive WM tracts. Meanwhile, compared with the Con group, the CSVD-D group shows significantly decreased FA values in a wide range of WM tracts. The extensive WM tracts described above included the anterior thalamic radiation, bilateral corticospinal tract, bilateral cingulate gyrus, bilateral hippocampus，forceps major, forceps minor, bilateral inferior fronto-occipital fasciculus, bilateral inferior longitudinal fasciculus, bilateral superior longitudinal fasciculus, bilateral uncinate fasciculus, and bilateral superior longitudinal fasciculus (temporal part) (FWE corrected, *p* < 0.05). However, there was no significant difference in MD values between CSVD-D group and Con group.

### Differences in structural network indicators

**Fig. 4:**
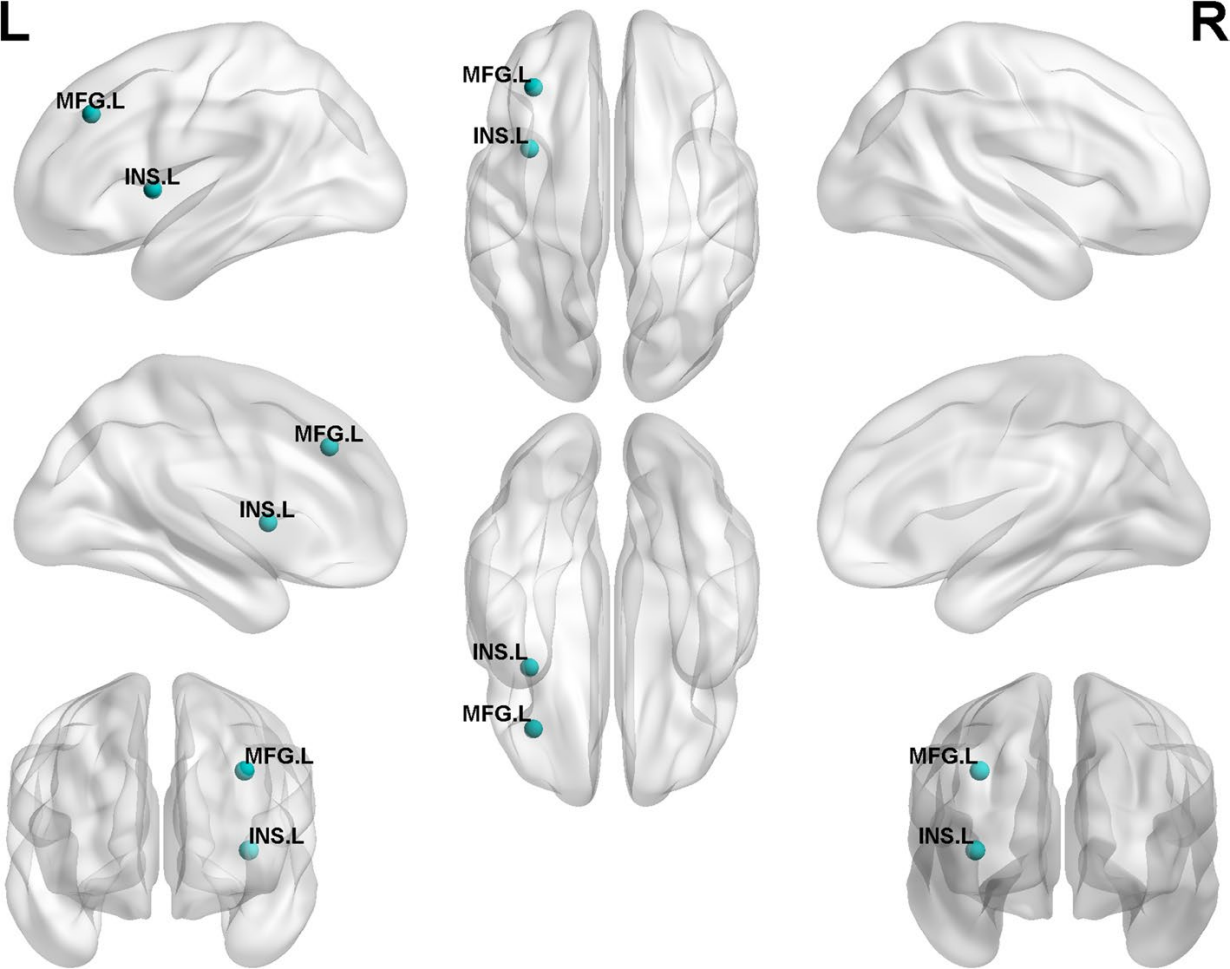
Regions with impaired regional efficiency in CSVD+D group relative to CSVD-D group. The regions impaired in efficiency are shown with blue nodes, with node size representing the between-group differences in regional efficiency (Bonferroni corrected, *p* < 0.05). Abbreviations: MFG. L represents left middle frontal gyrus, INS. L represents left Insula

**Fig. 5:**
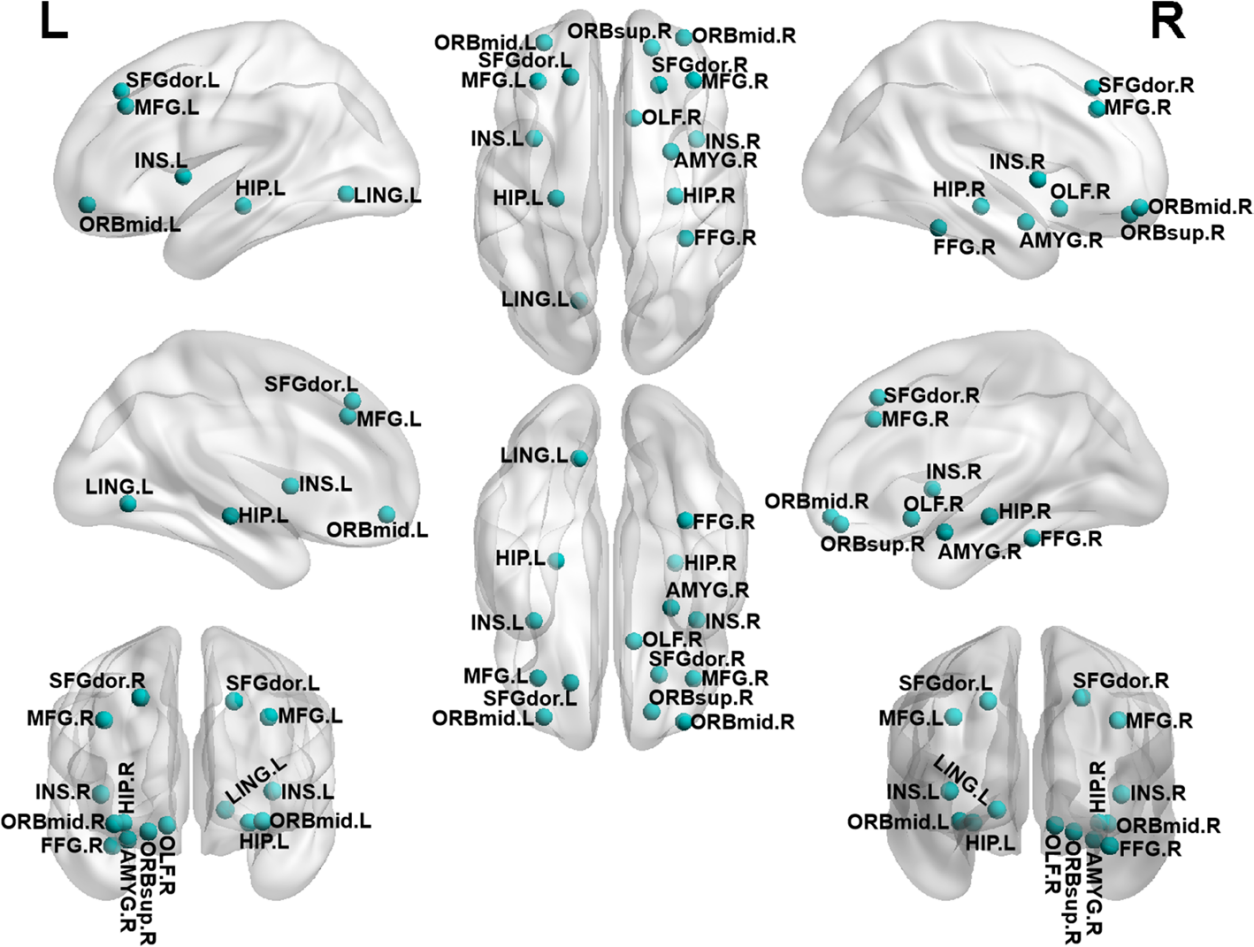
Regions with impaired regional efficiency in CSVD+D group relative to control group. The regions impaired in efficiency are shown with blue nodes, with node size representing the between-group differences in regional efficiency (Bonferroni corrected, *p* < 0.05). Abbreviations: MFG. L and MFG. R represents bilateral middle frontal gyrus, INS. L and INS. R represents bilateral lnsula, HIP. L and HIP. R represents bilateral hippocampus, ORBmid. L and ORBmid. R represents bilateral orbital middle frontal gyrus, SFGdor. L and SFGdor. R represents bilateral dorsolateral superior frontal gyrus, OLF. R represents right olfactory cortex, FFG. R represents right fusiform gyrus, LING. L represents left lingual gyrus, AMYG. R represents right amygdala, ORBsup.R represents right superior frontal gyrus of orbit

**Fig. 6:**
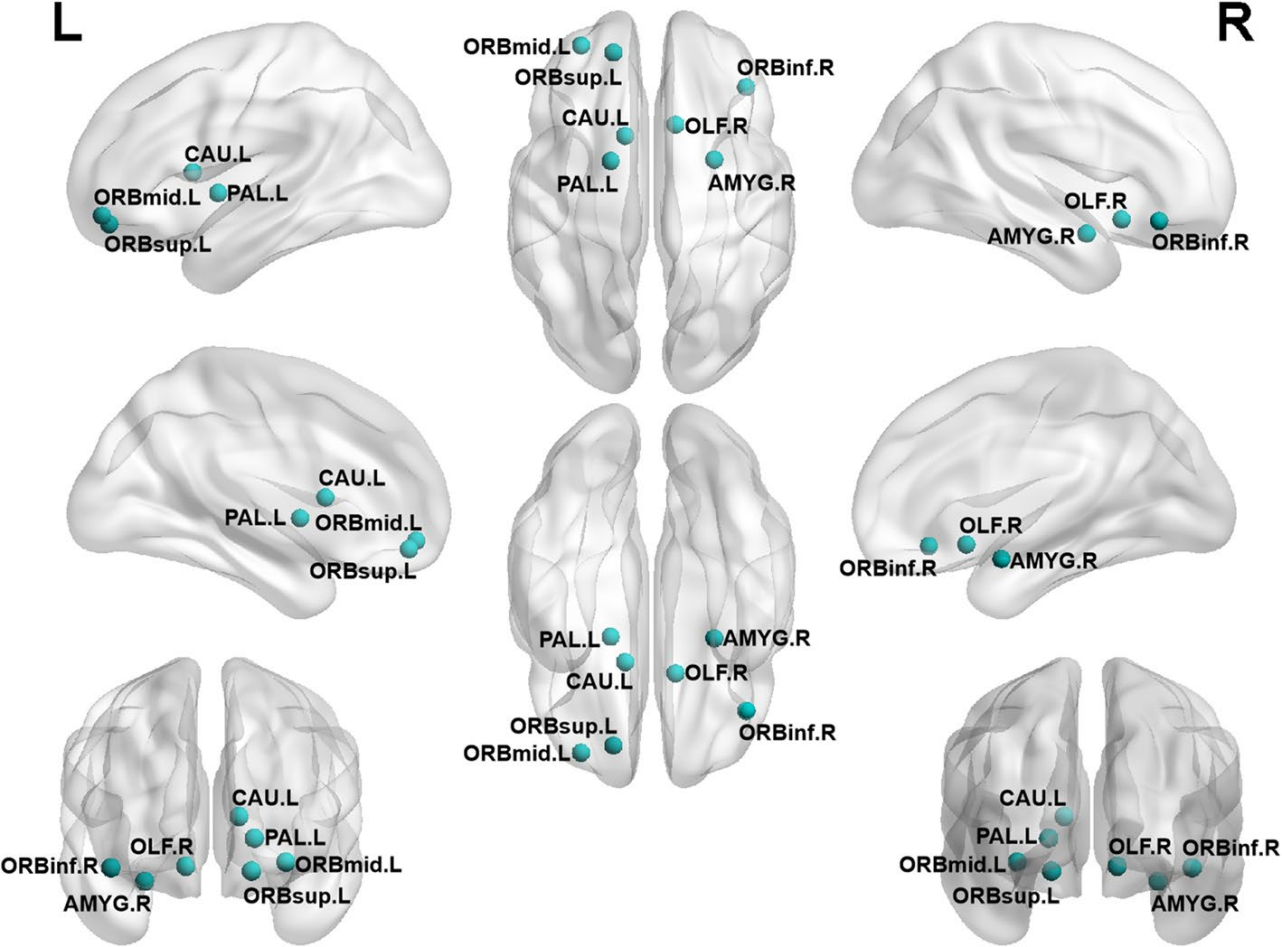
Regions with impaired regional efficiency in CSVD-D group relative to control group. The regions impaired in efficiency are shown with blue nodes, with node size representing the between-group differences in regional efficiency (Bonferroni corrected, *p* < 0.05). Abbreviations: CAU. L represents left caudate nucleus, PAL. L represents left lenticular globus pallidus, ORBmid. L represents left orbital middle frontal gyrus, AMYG. R represents right amygdala, ORBsup. L represents left superior frontal gyrus of orbit, ORBinf. R represents right lnferior frontal gyrus of orbit, OLF. R represents right olfactory cortex

The Con group, CSVD-D group, and CSVD+D group showed good small world properties at all thresholds (γ > 1, λ ≈ 1, σ > 1). We analyzed the differences in the structural network of the three groups, statistically significant results by Bonferroni correction. In Fig. 4, compared with CSVD-D group, CSVD+D group had obviously damaged nodes, mainly located in the left middle frontal gyrus (*p* = 0.006) and the left insula (*p* = 0.012). In Fig. 5, compared with the Con group, the nodes with significantly impaired efficiency in the CSVD+D group were mainly located in the bilateral hippocampus (left- *p* = 0.036, right- *p* = 0.001), bilateral middle frontal gyrus (orbital part) (left- *p* = 0.004, right- *p* = 0.021), bilateral middle frontal gyrus (left- *p* = 0.001, right- *p* = 0.024), bilateral insula (left- *p* = 0.001, right- *p* = 0.019), bilateral superior frontal gyrus (dorsolateral) (left- *p* = 0.033, right- *p* = 0.006), right olfactory cortex (*p* = 0.007), right fusiform gyrus (*p* = 0.007), right superior frontal gyrus (orbital part) (*p* = 0.019), right amygdala (*p* = 0.020) and left lingual (*p* = 0.011). In Fig. 6, compared with the Con group, the CSVD-D group had significantly impaired nodes, mainly located in the left caudate (*p* = 0.008), left pallidum (*p* = 0.010), left middle frontal gyrus (orbital part) (*p* = 0.011), left superior frontal gyrus (orbital part) (*p* = 0.021), right amygdala (*p* = 0.018), right inferior frontal gyrus (orbital part) (*p* = 0.023) and right olfactory cortex (*p* = 0.027).

### Correlation analysis

Partial correlation analysis was performed between DTI measures and depression scale scores after adjustment for age, gender, education and VRF. A significant correlation was found between FA and HAMD scores (*r* = − 0.291, *p* < 0.05), MD and HAMD scores (*r* = 0.297, *p* < 0.05), E_Local_ and HAMD scores (*r* = − 0.278, *p* < 0.01), network strength and HAMD scores (*r* = − 0.403, *p* < 0.001). However, no significant correlation was observed between E_Global_ and HAMD scores (*r* = − 0.142, *p* = 0.136).

## Discussion

There were no significant differences in terms of CSVD burden scores and conventional imaging findings between participants in the CSVD-D and CSVD+D groups, which indicates that conventional MRI findings may not be of much significance for the discovery of patients with CSVD complicated with depression, so further analysis of advanced MRI sequences, such as DTI, is required in the follow-up. We investigated DTI sequences based on TBSS analysis methods to identify damage to white matter fibers that occurs during the disease process. The results indicated that white matter fiber tracts of CSVD patients with depression were damaged. For FA and MD, compared with participants in the Con group and the CSVD-D group, participants in the CSVD+D group showed different degrees of damage to fiber tracts in almost all parts of the brain. Correlation analysis showed that FA was negatively correlated with HAMD scores, and MD was positively correlated with HAMD scores. Notably, there was no significant difference between participants in the Con group and CSVD-D group in terms of MD, which means that in patients with CSVD and depression, while demyelination of nerve fibers occurs, the microstructural organization is also significantly damaged. FA measures the restriction of the movement of water molecules in all directions and can describe the histomorphological features of the region, and decreased FA is associated with disturbed white matter fiber homogeneity [33]. Some studies reported that MD may be more sensitive in detecting mild damage, while FA captures more severe damage [34, 35]. Long associated fibers, such as the superior longitudinal fasciculus and uncinate fasciculus, showed a significant increase in MD in the preclinical stage. These long-associated fibers are generally considered to form myelin relatively late and are vulnerable to injury and decline in later life [36–38]. Disruption of white matter integrity may be related to changes in the structure and density of nerve fibers, as well as decreased axonal numbers, abnormal myelin sheaths, and gliosis [39]. This change may be the neuroanatomical basis of CSVD with depression, which reflects the hypothesis of “vascular depression” to a certain extent [40]. Hence, the damaged cingulate gyrus found in this study is an important part of the emotional circuit and participates in the processes of emotion and self-evaluation, which are closely related to depressive symptoms. Some scholars have found abnormalities in cerebral blood flow and metabolism in the posterior cingulate gyrus of patients with depression, which suggests that depression may correspond to low function of the posterior cingulate gyrus [41].

The significant finding of this study is that the characteristics of the structural network are closely related to depression in patients with CSVD. Because the structural brain network represents the integrity of white matter connections, it arguably reflects the underlying mechanisms of depression in patients with CSVD more than any other measure. The structural network connectivity of participants in the CSVD-D group was significantly higher than that of participants in the CSVD+D group. The present study found that with regard to regional efficiency, participants in the Con group showed significantly higher values than participants in the CSVD+D group in several areas, including the bilateral hippocampus, bilateral middle frontal gyrus (orbital part), bilateral middle frontal gyrus, bilateral insula, bilateral superior frontal gyrus (dorsolateral), right olfactory cortex, right fusiform gyrus, right superior frontal gyrus (orbital part), right amygdala and left lingual; most of these areas are part of the limbic system. The limbic cortex includes the allogenic cortex (such as the ancient cortex of the hippocampus and dentate gyrus, the septum and the amygdala, etc.) and the intermediate cortex (including the posterior part of the orbitofrontal cortex, insula, temporal pole, cingulate gyrus, etc.,), which participate in mediating instinctual and emotional behavior [42]. At the same time, neuroanatomy has confirmed that the prefrontal lobe is the key part of emotion regulation, which is related to cognition, emotion and conscious experience. The hippocampus is mainly responsible for memory and emotional control. Studies have shown that patients with recurrent and persistent depression have a significantly smaller hippocampus in the brain. However, no changes in this feature of the depression were found in the primary stage, which means that the disease itself caused this change in the brain. However, the study also pointed out that this change is reversible because the shrinkage is mainly due to the disconnection of nerve cells rather than the death of nerve cells, as in dementia. The combination of psychological and drug therapy can promote hippocampal regrowth [43]. We also studied non-depressed CSVD patients as well as healthy controls. The results showed that DTI measurements deteriorated gradually from Con to CSVD, followed by concomitant depression.

The above TBSS analysis results were highly consistent with the results of the structural network. A study found that the fiber tracts connected to the superior frontal gyrus included the inferior fronto-occipital fasciculus (IFOF), which connects to the lingual and gyrus parahippocampal/hooked cingulum [44]. These fiber bundles were closely related to the limbic system. In the insula, white matter fibers from the terminal capsule gather together and surround the uncinate fasciculus, radiating connections to the temporal pole, the medial temporal lobe and the amygdala complex [45]. The superior longitudinal fasciculus II is related to the connection between the angular gyrus and the anterior/ middle parts of the middle frontal gyrus [46]. The study found that FA values in the right SLF were positively correlated with the severity of anxiety and depression [47]. The uncinate tract is involved in the fiber connections of the entorhinal cortex, fusiform gyrus, superior temporal gyrus, and middle temporal gyrus [48]. The olfactory forebrain is closely related to the limbic system [49], the amygdala is a subcortical structure of the limbic system. It connects with the olfactory cortex through fibers and is involved in neural circuits of fear and defensive behavior [50].

Possible mechanisms of the pathogenesis of depressive symptoms in CSVD are as follows: 1. The “vascular depression hypothesis” holds that the increased incidence of depression in patients with CSVD may be due to the destruction of the subcortical pathway connecting brain regions, namely, cerebral white matter lesions which caused by cerebrovascular diseases, damage the the prefrontal subcortical circuit. It suggests that structural damage inherently disrupts network efficiency, leading to a depressive state [40, 51]. 2. CSVD is destructive when it occurs; it disrupts the corticostriatal-pallidum-thalamocortical circuit and affects noradrenergic and serotonergic neural pathways, resulting in a decrease in the levels of these two neurotransmitters. The subsequent corresponding metabolic activities are hindered, and the inability to achieve normal physiological activities results in depression [52]. 3. Cerebral ischemia leads to oxidative stress, and free radicals can attack the brain’s nerve tissue, cause astrocyte apoptosis, interfere with the maintenance of brain tissue and repairment of nerve injury by microglia, which is also an important cause of depression-related network damage [53, 54].

## Limitations

This study still had some limitations. First, the sample size of our study was relatively small, although we felt that the study was sufficiently effective for the analysis performed. Second, this was a cross-sectional cohort study, and we could not draw a causal relationship between depressive states and brain structural changes, as that would require repeated measures of longitudinal data. Third, this article only included patients aged 50–80 years, and CSVD patients of other age groups with depressive states require further study with large samples. Fourth, deterministic fiber tracking has some limitations in tracking complex white matter structures, especially cross fibers, which will be overcome by more advanced white matter imaging technology.

## Conclusion

In this study, DTI technology was applied to elderly patients with CSVD and depression. At the same time, the degree of depression was evaluated by the HAMD score, FA, and MD values; also, structural network indices in patients’ DTI parameters were effectively measured. Through data comparison and analysis, the results suggest that FA, MD and/or structural network indices change in emotion-related brain areas (the frontotemporal lobe, thalamus, cingulate gyrus and hippocampus), which revealed the integrity of brain white matter is damaged in CSVD patients with depression. The change in white matter microstructure and structural network index can reflect the degree of depression to a certain extent, providing a more direct structural basis for the hypothesis of abnormal neural circuits in the pathogenesis of vascular depression.

## Data Availability

The datasets generated and/or analyzed during the current study are not publicly available due to the complexity and uniqueness of the raw MRI data, as well as the limitations of laboratory policies but are available from the corresponding author on reasonable request.
